# LiDAR-registered freehand SPECT combined with NIR-fluorescence enables spatial localisation of tracer-avid inguinal lymph nodes: A clinical feasibility study

**DOI:** 10.1007/s00259-026-08075-y

**Published:** 2026-07-21

**Authors:** Rick W. A. Verdijk, Giusi Pisano, Matthias N. van Oosterom, Evie de Groot, Helena Zuurdeeg, Oscar R. Brouwer, Henk G. van der Poel, Fijs W. B. van Leeuwen

**Affiliations:** 1 Interventional Molecular Imaging Laboratory, Department of Radiology, Leiden University Medical Center, PO BOX 9600, Albinusdreef 2 (C2-S zone), 2300 RC Leiden, the Netherlands; 2 Department of Urology, Netherlands Cancer Institute-Antoni Van Leeuwenhoek Hospital, Amsterdam, the Netherlands; 3 Nuclear Medicine Unit, Department of Diagnostic Imaging and Oncological Radiotherapy, Fondazione Policlinico Universitario A. Gemelli IRCCS, Rome, Italy; 4 Department of Urology, Amsterdam University Medical Centres, VUmc, Amsterdam, the Netherlands

**Keywords:** Sentinel node, image-guided surgery, freehand SPECT, NIR-fluorescence, light detection and ranging (LiDAR)

## Abstract

**Purpose:**

Conventional ex vivo molecular assessment of nodal specimens lacks three-dimensional (3D) spatial context, making localisation of tracer-avid sentinel nodes (SNs) within large en bloc inguinal lymph node dissection (ILND) specimens challenging. We evaluated the technical feasibility of combining light detection and ranging (LiDAR)-registered freehand SPECT (*fh*SPECT) with near-infrared (NIR) fluorescence imaging for spatially augmented localisation of hybrid tracer uptake in solitary SNs and SNs residing within ILND specimens.

**Methods:**

Fourteen patients with penile cancer (25 cN0 groins; 3 cN1 groins) underwent radio- and fluorescence-guided lymph node (LN) surgery following preoperative lymphoscintigraphy and SPECT/CT. Resected specimens underwent conventional ex vivo assessment, followed by LiDAR-registered *fh*SPECT with co-registered NIR-fluorescence imaging. These 3D models were compared with conventional ex vivo assessments, preoperative imaging, and histopathological outcomes.

**Results:**

In total, 40 solitary tracer-avid LNs and 3 ILND specimens underwent ex vivo assessment. LiDAR/*fh*SPECT localised radioactive hotspots in 38/40 solitary LNs, consistent with conventional gamma probe assessment, while both conventional and LiDAR-registered NIR-fluorescence imaging detected fluorescent signal in all 40 LNs. Among the 3 ILND procedures, preoperative SPECT/CT identified 6 tracer-avid hotspots within the anatomical resection template. Conventional gamma probe and NIR-fluorescence detected only 4 and 5 hotspots, respectively, whereas LiDAR/*fh*SPECT localised all 6 hotspots in the corresponding resected specimens. Histopathology identified 6 tumour-positive LNs, all of which were hybrid tracer-avid and effectively localised by LiDAR/*fh*SPECT.

**Conclusion:**

LiDAR-registered 3D surface mapping provided anatomical context to radioactive and fluorescent molecular imaging signals, enabling localisation of tracer-avid LNs in complex ex vivo ILND specimens. These findings support the feasibility of spatially augmented localisation of hybrid tracer uptake and warrant validation in larger studies.

## Introduction

Inguinal lymph node (LN) involvement is a crucial prognostic factor for survival in patients with penile squamous cell carcinoma (SCC) [1]. Accurate nodal assessment in patients at intermediate- to high risk of LN metastasis (primary tumour stage ≥T1bG2) is therefore considered essential for disease staging, risk stratification, and treatment planning. Sentinel nodes (SNs), representing the first lymph node(s) draining from the primary tumour site, are the most likely sites of early nodal metastatic spread [3]. In penile cancer, SNs are typically located in the inguinal region.

In clinically tumour-negative (cN0) groins, the minimally invasive SN procedure is the preferred surgical staging technique [2, 3], enabling early detection of nodal metastasis [4, 5]. In contrast, clinically tumour-positive groins (cN1) – which may occur concurrently within an opposite groin of the same patient – require an invasive radical inguinal LN dissection (ILND). ILND provides maximal oncological control but is associated with substantial morbidity due to disruption of the lymphatic anatomy [6].

In some high-volume penile cancer centres, the SN procedure is performed using a hybrid radioactive and fluorescent tracer (indocyanine green (ICG)-^99m^Tc-Nanocolloid or ICG-^99m^Tc-Nanoscan [7, 8]). These hybrid tracers enable preoperative identification of SNs using lymphoscintigraphy and single-photon emission computed tomography (SPECT/CT) and facilitate intraoperative targeted excision guided by a handheld gamma probe and near-infrared (NIR) fluorescence imaging [9]. To verify completeness and accuracy of nodal harvesting, intraoperative SN detection is routinely confirmed by ex vivo assessment of the resected LNs [7]. At present, confirmation of SN removal within ILND specimens is uncommon, primarily because overlying tissue can obscure the distinction between adjacent radioactive hotspots or attenuate fluorescent signals from deeper-lying nodes. Failure to identify SNs within larger ILND specimens may introduce spatial ambiguity, potentially affecting confidence in oncological completeness and pathological sampling.

More fundamentally, although intraoperative molecular imaging techniques reliably detect tracer uptake, they generally lack 3D anatomical context. This limitation could be addressed by 3D intraoperative imaging modalities compatible with the dynamic surgical environment.

Freehand (*fh*)SPECT is an intraoperative molecular imaging modality that enables real-time, gantry-free 3D reconstruction of radiotracer distribution through spatial tracking of a handheld gamma detector during acquisition [10–15]. Unlike conventional gamma probe assessment, which provides point-based acoustic and quantitative feedback through count-rate measurements (counts per second [cps]; dimensionless), *fh*SPECT enables 3D visualisation of radiotracer uptake. Initially, *fh*SPECT data were displayed as a 2D augmented reality overlay on a conventional video feed. More recently, *fh*SPECT has been combined with light detection and ranging (LiDAR), a scanning technology that generates high-resolution 3D surface reconstructions using reflected light signals, creating a so-called spatial template of the target. 3D surface scanning is increasingly being used in clinical workflows requiring accurate anatomical reconstructions, including dentistry, maxillofacial surgery, and orthopaedics [16–19]. Within the molecular imaging field, integration of LiDAR with *fh*SPECT enables co-registration of the 3D radiation map with the morphology of resected specimens, creating a spatially corrected augmented reality overlay. This concept has previously been assessed in PSMA-avid prostate cancer specimens [20, 21], but has not yet been evaluated for the assessment of SN and ILND specimens. In addition, its integration with NIR-fluorescence imaging has not previously been described.

The aim of this study was to evaluate whether ex vivo LiDAR-registered *fh*SPECT and NIR-fluorescence imaging could provide spatially augmented localisation of hybrid tracer uptake in isolated tracer-avid SN specimens and SNs within larger ILND specimens obtained from penile cancer patients.

## Methods

### Patients and study design

Patients with penile SCC and a primary tumour at intermediate- to high-risk for nodal metastasis (≥ T1bG2) undergoing radio- and fluorescence-guided LN surgery between September and December 2025 were eligible for inclusion. SN procedures were performed in groins with clinically node-negative (cN0) inguinal lymph nodes, as established by ultrasound and ultrasound-guided fine-needle aspiration cytology (FNAC). In groins with cytologically proven lymph node metastases (cN1), ILND was performed. In these patients, preoperative [¹⁸F]FDG positron emission tomography/computed tomography (PET/CT) was performed to exclude pelvic nodal or distant metastatic disease. Patients with pelvic nodal or distant metastatic disease were excluded because they required alternative treatment strategies.

This prospective feasibility study was approved by the local institutional review board (IRBdm24-249).

### Preoperative and in vivo workflow

All patients received a peritumoural injection (4 deposits) of the hybrid tracer ICG–⁹⁹ᵐTc-Nanoscan prior to surgery as standard of care [22]. The mean injected activity of ICG-^99m^Tc-Nanoscan was 104.9 MBq (range, 99–112 MBq) for the 1-day protocol (injection and surgery on the same day) and 212 MBq (range, 203–221 MBq) for the 2-day protocol (injection the day before surgery).

Preoperative lymphoscintigraphy was performed to identify the presence, number, and anatomical distribution of SNs per groin. Following dynamic, early, and delayed planar imaging, SPECT/CT (Symbia Intevo Bold; Siemens Healthineers, Erlangen, Germany) was performed to enable detailed anatomical localisation of the SNs for surgical planning [23].

Intraoperatively, suspected hybrid tracer-avid lymph nodes were localised using a handheld gamma probe (Neoprobe; Navidea Biopharmaceuticals, Dublin, OH, USA) in combination with NIR-fluorescence imaging (CE-marked FIS-00 prototype; Hamamatsu Photonics, Hamamatsu, Japan).

### Conventional ex vivo assessment

Immediately after excision, ex vivo radioactive count rates were measured using the handheld gamma probe, while NIR-fluorescence intensity was qualitatively assessed using the FIS-00 camera and classified as “*bright*”, “*moderate*”, “*weak*”, or “*absent*”. For resected ILND specimens, the number and anatomical distribution of radioactive and fluorescent hotspots were recorded, including radioactive count rates and fluorescence intensity measurements. Specimens were then transferred for subsequent LiDAR-based ex vivo assessment.

### LiDAR-registered ex vivo measurements

**Fig. 1:**
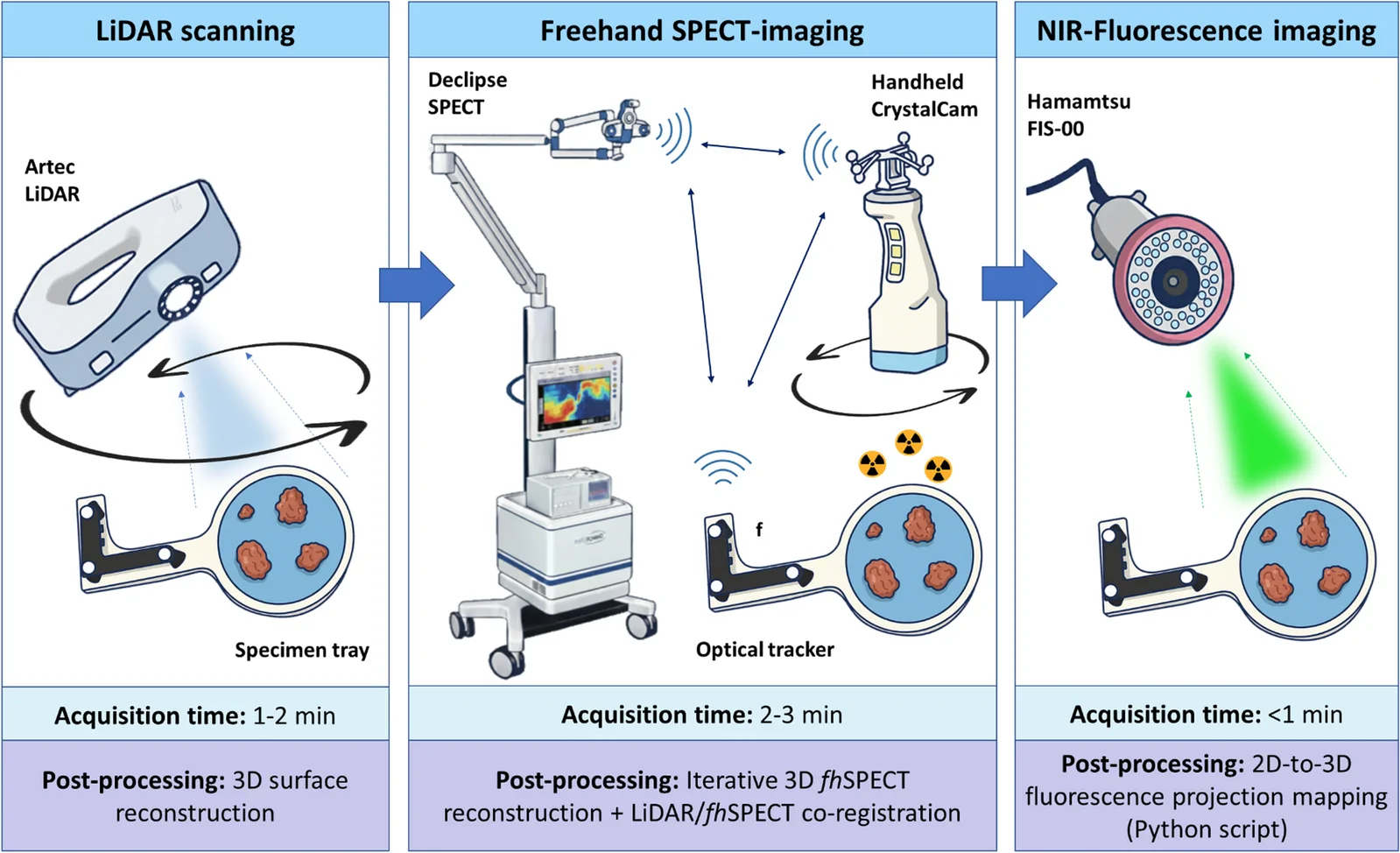
Ex vivo LiDAR-registered 3D *fh*SPECT and 2D NIR-fluorescence imaging workflow. Specimens were positioned on a custom-built specimen tray equipped with integrated optical tracking markers. A high-resolution 3D reconstruction of the target objects was acquired using the Artec Light Detection and Ranging (LiDAR) system, which emits high-frequency light pulses and reconstructs specimen geometry based on the reflected light (acquisition time: 1–2 min). Subsequently, the Declipse SPECT system was used to perform a freehand SPECT (*fh*SPECT) by spatially tracking the handheld gamma detector (CrystalCam) in real time, allowing reconstruction of the 3D radiotracer distribution within the region of interest (acquisition time: 2–3 min). Finally, near-infrared (NIR)-fluorescence imaging was performed using the Hamamatsu FIS-00 camera system to obtain 2D fluorescence images (acquisition time: <1 min). Post-processing included automated LiDAR/fhSPECT co-registration, manual fiducial-based NIR-fluorescence registration, and hotspot visualisation using interactive threshold adjustment (3D Slicer software), resulting in a total acquisition and processing time of approximately 10–12 min per specimen tray

3D surface scanning was performed using the workflow as previously described [20, 21]. Resected SN and ILND specimens were placed on a custom-built specimen tray equipped with an integrated reference tracker to enable accurate spatial registration. A handheld LiDAR scanner (Artec Eva; Artec3D, Luxembourg) was used to generate high-resolution 3D surface reconstructions of the specimens [24, 25]. Acquisition time for the 3D surface scan was approximately 1–2 min per specimen tray (Fig. 1).

*fh*SPECT was performed to visualise radioactive tracer distribution within resected specimens using the mobile declipseSPECT system (SurgicEye GmbH, Berlin, Germany) in combination with an optically tracked handheld gamma camera (CrystalCam; Crystal Photonics GmbH, Berlin, Germany) [26, 27]. After a 2–3-minute acquisition, a 3D *fh*SPECT reconstruction was generated using an iterative maximum-likelihood expectation maximization algorithm (20 iterations) with isotropic voxels and a pixel spacing of 3 mm.

Subsequently, NIR-fluorescence imaging was performed using the FIS-00 camera system (acquisition time < 1 min) to visualise tracer distribution within the specimens based on the fluorescent ICG signal.

### Multimodal spatial co-registration and augmented reality reconstruction

LiDAR-registered *fh*SPECT spatial augmented reality reconstructions were generated by fusing the high-resolution 3D surface scan of the specimen (OBJ format), acquired by LiDAR scanning, with the corresponding 3D *fh*SPECT dataset (DICOM format). Both imaging acquisitions were performed while the specimens remained fixed on the same optically tracked specimen tray, preserving a shared spatial coordinate system throughout acquisition. This enabled automated co-registration of the LiDAR-derived anatomical surface model with the *fh*SPECT-derived radioactive signal distribution without requiring specimen repositioning. Visualisation and analysis of the fused datasets were performed in 3D Slicer (version 5.6.2).

No predefined quantitative threshold for hotspot detection was applied. Instead, interactive threshold adjustment was performed by a single experienced operator based on visual assessment of hotspot visibility relative to the surrounding background activity. Signal scaling was adjusted to optimise hotspot visualisation by balancing background suppression, facilitating separation of adjacent hotspots, and preserving the detection of low-intensity focal uptake.

**Fig. 2:**
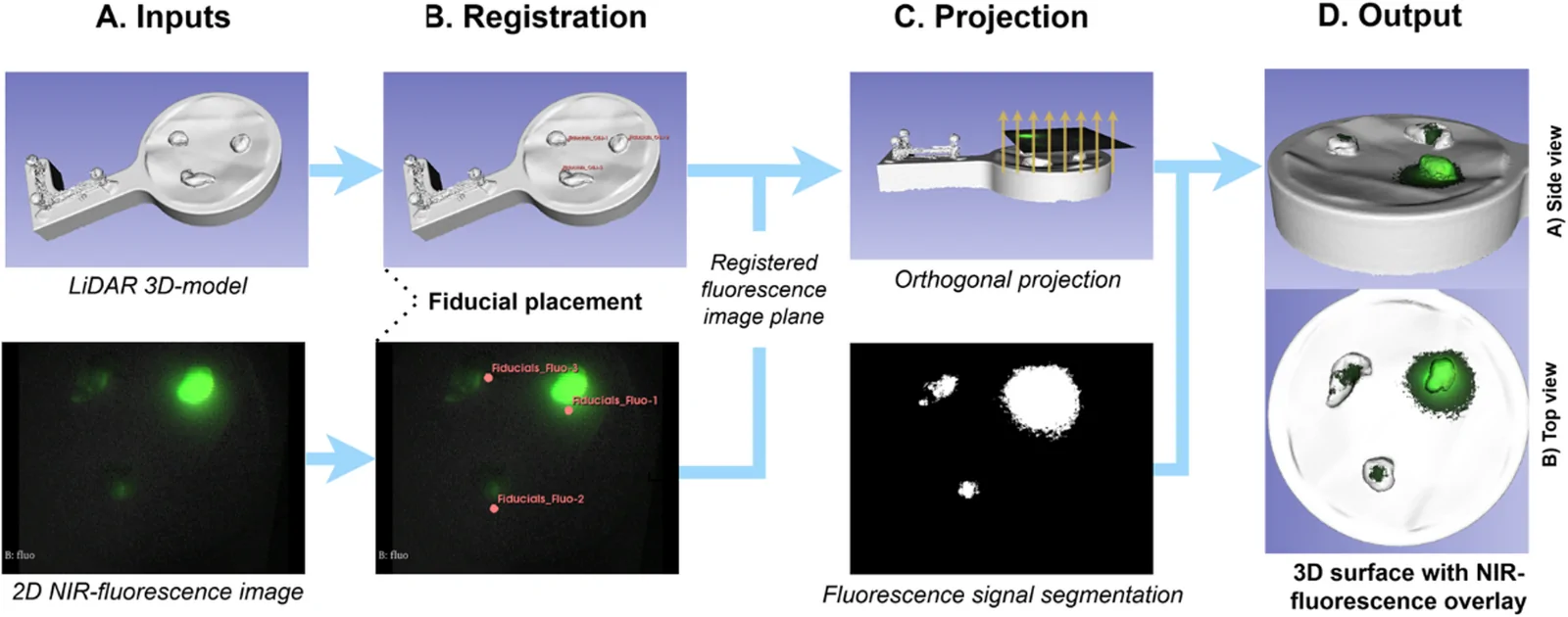
Spatially augmented LiDAR/NIR-fluorescence imaging. A 3D surface model of the specimen tray was acquired using handheld LiDAR scanning (top), and the corresponding 2D near-infrared (NIR) fluorescence molecular imaging data of the specimen tray was acquired using the Hamamatsu FIS-00 (bottom) (**A**). Fiducial markers were manually placed on corresponding anatomical landmarks within the 3D surface model and 2D NIR-fluorescence image, to establish spatial correspondence and facilitate accurate alignment onto the 3D geometry (**B**). The registered 2D NIR-fluorescence image plane was orthogonally projected onto the 3D surface scan using a custom Python script (top). Fluorescence signal was segmented (Hue-Saturation-Value (HSV) segmentation) from the background to isolate regions of interest (bottom) (**C**). The 3D surface model overlaid with segmented NIR-fluorescent signal enables spatial registration onto the 3D anatomical structure of the resected specimens (**D**)

Because the FIS-00 fluorescence camera did not provide native 3D spatial tracking, LiDAR/NIR-fluorescence co-registration required a separate manual registration step. Using a custom Python script implemented in the 3D Slicer environment, 2D fluorescence images were segmented and projected onto the LiDAR-derived 3D surface mesh using manually selected corresponding anatomical fiducial landmarks (Fig. 2). The total acquisition, processing, and registration time was approximately 10–12 min per specimen tray, excluding specimen transfer from the operating room.

### Experimental groups and outcome definitions

Solitary resected tracer-avid lymph nodes were used to assess the technical feasibility and exploratory localisation agreement of the multimodal imaging workflow. Because these specimens could be evaluated individually, agreement between LiDAR-registered and conventional imaging modalities was assessed on a per-lymph node basis, using intraoperative tracer-avid lymph node detection (gamma probe and/or NIR-fluorescence) as the reference standard.

The potential added value of spatially co-registered 3D models for localising tracer-avid hotspots within ILND specimens was further explored. Because en bloc ILND specimens precluded direct node-by-node correlation during ex vivo assessment, tracer-avid hotspots identified on preoperative SPECT/CT served as the anatomical reference framework for hotspot localisation. This approach enabled comparison of the expected number and anatomical distribution of tracer-avid hotspots.

### Pathological analysis

Following ex vivo specimen scanning, all resected LN specimens underwent routine pathological processing, including haematoxylin and eosin (H&E) staining. Within ILND specimens, the locations of radioactive and fluorescent hotspots were marked using reference stitches to support pathological correlation. Pathological assessment determined the presence or absence of metastatic disease within harvested SNs or ILNDs. Histopathological assessment was used solely for tumour identification and not as a reference standard for tracer-avidity within resected specimens.

### Statistical analysis

Given the exploratory nature and limited sample size, no formal power calculation was performed.

Exploratory descriptive performance metrics (sensitivity, specificity, accuracy, and inter-modality concordance) were calculated within the applied reference frameworks and should be interpreted descriptively given the sample size and differing reference standards.

Patient, surgical, and specimen characteristics were summarised using descriptive statistics and are reported as mean with standard deviation (SD), median with interquartile range (IQR), or proportions, as appropriate.

Associations between ex vivo radioactive cps and quantified NIR-fluorescence intensity were explored. Statistical significance was defined as *p* < 0.05. All statistical analyses were performed using R software, version 4.4.2.

## Results

### Patient cohort and specimen characteristics

**Fig. 3:**
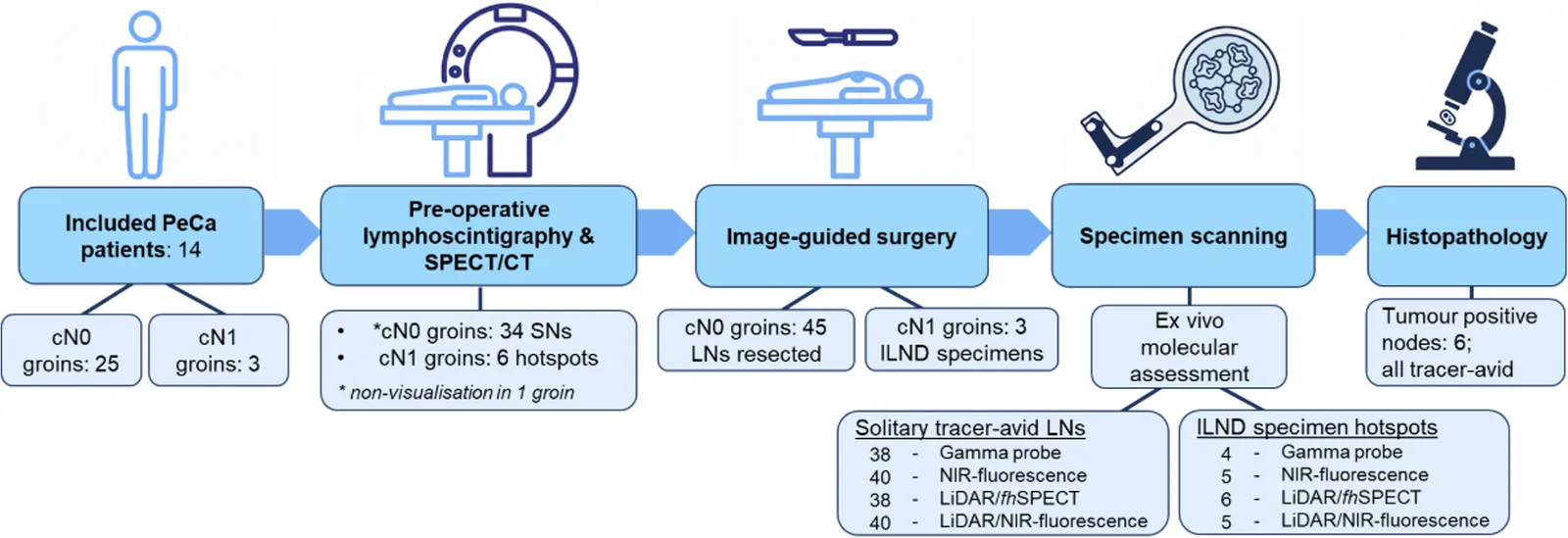
Study workflow and key findings. Eligible penile cancer (PeCa) patients with clinically node-negative (cN0) or clinically node-positive (cN1) groins underwent preoperative lymphoscintigraphy and SPECT/CT, image-guided surgery, ex vivo molecular assessment, and histopathological evaluation. The figure summarises the number of detected sentinel nodes (SNs), tracer-avid hotspots, resected specimens, and tumour-positive lymph nodes across the study workflow

A total of 14 penile cancer patients undergoing hybrid tracer-guided LN surgery were prospectively included (Table 1; Fig. 3). Bilateral SN procedures (cN0 in both groins) were performed in 11 patients, whereas the remaining 3 patients underwent ILND in the tumour-affected (cN1) groin combined with contralateral SN sampling in the tumour-negative (cN0) groin. Median age was 70 years (range 53–83), and median BMI was 29 (range 23–47). Primary tumour stage ranged from cT1b to cT3.

**Table 1:** Baseline characteristics. For each case, this table reports preoperative sentinel node identification, surgical approach, radioactive and fluorescent nodal detection, the number and type of specimens assessed using LiDAR-registered freehand SPECT (*fh*SPECT) and LiDAR-registered NIR-fluorescence imaging, and the concordance between the two hybrid imaging modalities for each groin

Case			Imaging	Surgery	Conventional ex vivo assessment			LiDAR/ fhSPECT ^X^	LiDAR/NIR- fluorescence ^Y^		Pathology	
	cTNM-stadium	Type of Surgery	SNs on SPECT/CT	Resected targets	Gamma probe ^A^ *n* (%)	FIS-00 ^B^ *n* (%)	Modality agreement ^A^ vs. ^B^	Specimens scanned (hotspots)	Specimens scanned (hotspots)	Modality agreement ^X^ vs. ^Y^	LNs found	Metastasis (mm)
1	T1b cN0	SN left SN right	3 1	2 SNs 1 SN	2 (100) 1 (100)	2 (100) 1 (100)	+ +	2 SNs (2) 1 SN (1)	2 SNs (2) 1 SN (1)	+ +	2 1	- -
2	T2 cN1	SN left ILND right	1 1	1 SN 1 ILND	1 (100) 1 (100)	1 (100) 1 (100)	+ +	1 SN (1) 1 ILND (1)	1 SN (1) 1 ILND (1)	+ +	1 6	1 (0.8) 1 (18)
3	T3 cN0	SN left SN right	1 1	1 SN 1 SN	1 (100) 1 (100)	1 (100) 1 (100)	+ +	1 SN (1) 1 SN (1)	1 SN (1) 1 SN (1)	+ +	1 1	- 1 (5)
4	T2 cN0	SN left SN right	1 1	1 SN 1 SN	1 (100) 1 (100)	1 (100) 1 (100)	+ +	1 SN (1) 1 SN (1)	1 SN (1) 1 SN (1)	+ +	1 1	- -
5	T3 cN0	SN left SN right	2 2	3 LNs 4 LNs	3 (100) 4 (100)	3 (100) 4 (100)	+ +	3 LNs (3) 4 LNs (4)	3 LNs (3) 4 LNs (4)	+ +	3 4	- -
6	T1b cN0	SN left SN right	1 2	2 LNs 2 SNs	1 (50) 2 (100)	1 (50) 2 (100)	+ +	2 LNs (1) 2 SN (1)	2 LNs (1) 2 SN (1)	+ +	2 2	- -
7	T2 cN0	SN left SN right	- 3	1 LN 3 SNs	1 (100) 3 (100)	1 (100) 3 (100)	+ +	1 LN (1) 3 SNs (3)	1 LN (1) 3 SNs (3)	+ +	1 3	- -
8	T2 cN0	SN left SN right	1 1	1 SN 2 LNs	1 (100) 1 (50)	1 (100) 2 (100)	+ -	1 LN (1) 2 LNs (1)	1 LN (1) 2 LNs (2)	+ -	1 2	- -
9	T2 cN0	SN left SN right	1 1	2 LNs 1 SN	1 (50) 1 (100)	1 (50) 1 (100)	+ +	2 LNs (1) 1 SN (1)	2 LNs (1) 1 SN (1)	+ +	2 1	- -
10	T3 cN1	SN left ILND right	1 3	1 SN 1 ILND	1 (100) 2 (66.7)	1 (100) 3 (100)	+ +	1 SN (1) 1 ILND (3)	1 SN (1) 1 ILND (3)	+ +	1 9	- 1 (19)
11	T3 cN0	SN left SN right	1 1	1 SN 2 SNs	1 (100) 1 (50)	1 (100) 1 (50)	+ +	1 SN (1) 2 LNs (1)	1 SN (1) 2 LNs (1)	+ +	1 2	- -
12	T2 cN0	SN left SN right	1 2	3 LNs 4 LNs	1 (33.3) 3 (75)	1 (33.3) 4 (100)	+ -	3 LNs (1) 4 LNs (3)	3 LNs (1) 4 LNs (4)	+ -	3 4	- -
13	T2 cN0	SN left SN right	1 3	1 SN 3 SNs	1 (100) 3 (100)	1 (100) 3 (100)	+ +	1 SN (1) 3 SNs (3)	1 SN (1) 3 SNs (3)	+ +	1 3	- -
14	T3 cN1	SN left ILND right	1 2	1 SN 1 ILND	1 (100) 1 (50)	1 (100) 1 (50)	+ +	1 SN (1) 1 ILND (2)	1 SN (1) 1 ILND (1)	+ -	1 10	- 2 (13, 18)

*SN* sentinel node, *LN* lymph node, *ILND* inguinal lymph node dissection, *fh* freehand, *SPECT* single−photon emission computed tomography, *LiDAR* Light Detection and Ranging, *FIS−00* fluorescence camera

### Preoperative imaging

**Fig. 4:**
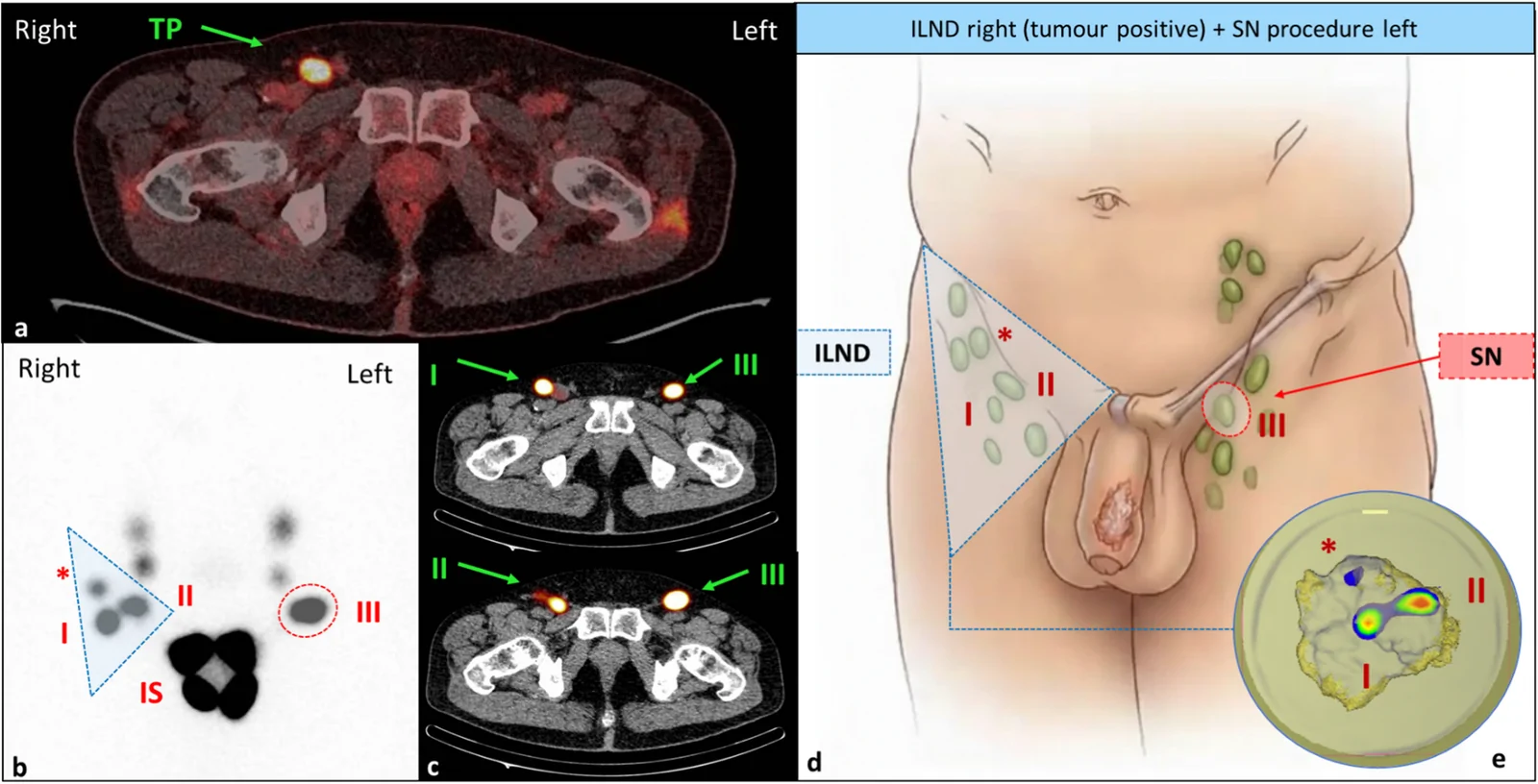
Preoperative imaging of a representative patient undergoing combined sentinel node biopsy and inguinal lymph node dissection. Following ultrasound and fine-needle aspiration cytology (FNAC) confirmation of metastatic involvement of a right inguinal lymph node, preoperative [¹⁸F]FDG-PET/CT (**a**) demonstrated the corresponding suspicious inguinal lymph node in the right groin (TP), without pelvic or distant metastasis. The patient was scheduled for an ILND procedure in the tumour-affected groin (cN1), and a SN procedure in the contralateral groin (cN0). Preoperative lymphoscintigraphy (**b**) showed the tracer injection site (IS), bilateral lymphatic drainage to SNs (I-III), and a higher-echelon node (HE, *) within the inguinal region. The four additional tracer-avid hotspots in the background correspond to HEs in the pelvic area. SPECT/CT (**c**) provided anatomical context to support surgical planning, and identified two SNs (I and II) and one HE (*) within the ILND resection template (**d**). Ex vivo LiDAR/*fh*SPECT provided spatial localisation of all tracer-avid nodes within the ILND specimen (**e**)

Within the 25 tumour-negative (cN0) groins, preoperative lymphoscintigraphy and SPECT/CT visualised 34 tracer-avid hotspots assigned as SNs, corresponding to a mean of 1.4 SNs per groin (range 1–3; Table 2). Despite reinjection of the hybrid tracer, preoperative non-visualisation occurred in one groin (case 7, left groin; Table 1). In the 3 tumour-positive (cN1) groins, 6 tracer-avid hotspots were identified within the anatomical ILND resection template (representative case shown in Fig. 4).

**Table 2:** Overview of surgical specimens and ex vivo multimodal imaging findings in the sentinel node (SN) exploratory feasibility cohort and inguinal lymph node dissection (ILND) exploratory implementation cohort. For solitary resected lymph nodes, intraoperative hybrid-tracer detection served as the applied reference framework for exploratory performance assessment. For ILND specimens, preoperative SPECT/CT hotspot localisation served as the anatomical reference framework for descriptive comparison of hotspot detection

(A) Sentinel node (SN) specimens – exploratory feasibility cohort		
Patients	Number of patients (groins)	14 (25)
Preoperative imaging	Tracer-avid LNs defined as SNs identified on preoperative SPECT/CT - SNs per groin, mean (range)	34 1.4 (1–3)
Surgical yield	Total excised LN specimens - Tracer-avid LNs (reference framework) - Non-tracer-avid LNs	45 40 5
* Conventional assessment*: Gamma probe	Tracer-avid LNs detected - Count rate (cps), mean (IQR)	38 5,531 (575–5,800)
*Conventional assessment*: NIR-fluorescence	Tracer-avid LNs detected - Fluorescence intensity grading	40 Bright: 27 Moderate: 6 Weak: 7
*LiDAR-registered assessment*: LiDAR/*fh*SPECT	Tracer-avid LNs detected, n/reference Non-tracer-avid LNs detected Exploratory performance estimates: - Sensitivity (38/40) - Specificity (5/5) - Accuracy (38 + 7)/45	38/40 5/5 95% 100% 96%
*LiDAR-registered assessment*: LiDAR/NIR-fluorescence	Tracer-avid LNs detected, n/reference Non-tracer-avid LNs detected Exploratory performance estimates: - Sensitivity (40/40) - Specificity (5/5) - Accuracy (40 + 5)/45	40/40 5/5 100% 100% 100%
Inter-modality agreement	Gamma probe vs. LiDAR/*fh*SPECT NIR-fluorescence vs. LiDAR/NIR-fluorescence	100% 100%
Pathology	LNs retrieved at pathology - Tumour-positive LNs, n (%) - Metastasis diameter (mm), mean (range)	45 2 (4.4%) 2.9 (0.8–5)
(B) Inguinal lymph node dissection (ILND) specimens** -** exploratory implementation cohort		
Patients	Number of patients (groins)	3 (3)
Preoperative imaging	Tracer-avid hotspots identified on preoperative SPECT/CT (reference framework) (mean per patient)	6 (2)
Surgical yield	Total excised ILND specimens	3
* Conventional assessment*: Gamma probe	Tracer-avid hotspots localised - Mean count rate, cps (IQR)	4/6 (66.7%) 716 (440–950)
* Conventional assessment*: NIR-fluorescence	Tracer-avid hotspots localised - Fluorescence intensity grading	5/6 (83.3%) Bright: 2 Moderate: 3
*LiDAR-registered assessment*: LiDAR/*fh*SPECT	Tracer-avid hotspots localised, n/reference	6/6
*LiDAR-registered assessment*: LiDAR/NIR-fluorescence	Tracer-avid hotspots localised, n/reference	5/6
Inter-modality agreement	Gamma probe vs. LiDAR/*fh*SPECT NIR-fluorescence vs. LiDAR/NIR-fluorescence	66.7% 100%
Pathology	LNs retrieved at pathology - Tumour-positive LNs, n (%) - Metastasis diameter (mm), mean (range)	25 4 (16%) 17 (13–19)

*SN* sentinel node, *LN* lymph node, *ILND* inguinal lymph node dissection, *fh* freehand, *SPECT* single−photon emission computed tomography, *LiDAR* Light Detection and Ranging, *NIR* near−infrared, *IQR* interquartile range

### Surgical yield

Surgical exploration during the SN procedure in 25 groins yielded 45 resected LNs. Of these, 40 (89%) were classified as tracer-avid; 38 were detected intraoperatively by both gamma tracing and NIR-fluorescence imaging, whereas 2 were identified exclusively by NIR-fluorescence imaging (Fig. 3). The remaining 5 resected LNs were not detected by either modality and were therefore classified as non-tracer-avid nodes (non-SNs). In the one groin without preoperative visualisation (case 7, left groin; Table 1), surgical exploration yielded one LN with low-level tracer avidity.

Of the 40 resected tracer-avid LNs, 33 corresponded to SNs identified on preoperative imaging. One preoperatively identified SN could not be localised intraoperatively and was therefore not resected (case 1, left groin; Table 1). The remaining 7 tracer-avid LNs corresponded to higher-echelon (HE) nodes identified on preoperative imaging.

During ILND in the 3 cN1 groins, nodal specimens were removed according to the standard surgical template, including en bloc dissection of all superficial and deep inguinal LNs within the femoral triangle [2].

### Conventional ex vivo assessment

#### Solitary LN specimens (cN0 groins)

Ex vivo gamma probe assessment detected radioactive signal in 38 of the 40 tracer-avid LNs. The mean count rate was 5,531 cps (IQR, 575–5,800). Fluorescence was observed in all 40 nodes and was qualitatively assessed as “*bright”* (68%), “*moderate*” (15%), or “*weak*” (18%) (Table 2). For both modalities, ex vivo findings were concordant with the intraoperative observations in all nodes. This included the two intraoperatively detected fluorescence-only nodes, which likewise lacked detectable radioactive signal on ex vivo gamma probe assessment.

Ex vivo assessment of the LN harvested from the non-visualised groin demonstrated a radioactive count rate of < 10% relative to the hottest node in the contralateral groin, together with weak fluorescence intensity, which may explain the failed preoperative visualisation.

#### ILND specimens (cN1 groins)

In the 3 ILND specimens, gamma probe assessment detected 4 hotspots (mean 716 cps, IQR 440–950), while NIR-fluorescence visualised 5 fluorescent hotspots (2 “*bright*”, 3 “*moderate*”) (Table 2).

In the first ILND specimen (case 2), the single tracer-avid hotspot identified on preoperative SPECT/CT was detected by both ex vivo modalities. In the second ILND specimen (case 10), all 3 preoperatively identified hotspots were visualised by NIR-fluorescence imaging, whereas gamma tracing distinguished only 2 hotspots. In the third ILND specimen (case 14), 2 tracer-avid hotspots were expected based on preoperative SPECT/CT, but only one could be clearly detected using either gamma tracing or NIR-fluorescence imaging (Table 1).

No significant association was observed between ex vivo gamma count rates and qualitative fluorescence intensity in solitary LNs and ILND specimens (Kruskal–Wallis, *p* = 0.587).

### LiDAR-registered ex vivo assessment

#### Solitary LN specimens (cN0 groins)

**Fig. 5:**
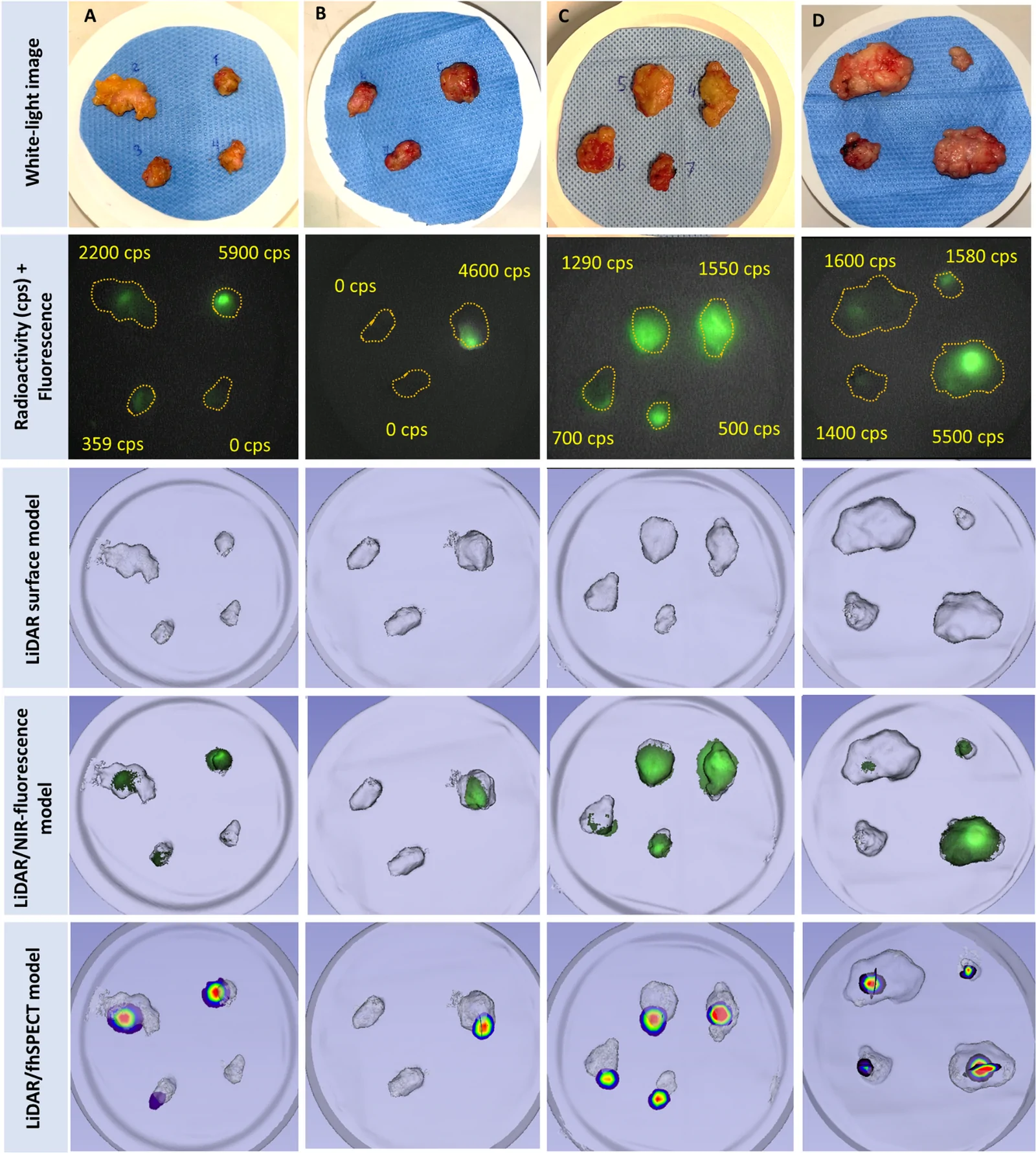
Ex vivo spatial concordance of tracer-avid lymph node localisation using LiDAR-registered *fh*SPECT and NIR-fluorescence imaging. Representative excised solitary lymph node specimens retrieved during the SN procedure are shown to illustrate concordant hybrid-tracer localisation across ex vivo imaging modalities (columns A–D). **A**: LiDAR/*fh*SPECT visualised all tracer-avid nodes across a broad spectrum of ex vivo radioactive counts (range, 359–5900 cps), with *fh*SPECT hotspot intensity qualitatively corresponding to relative gamma probe signal intensity. **B**: Two non-sentinel nodes without detectable LiDAR/*fh*SPECT or LiDAR/NIR-fluorescence signal. **C**: Four tracer-avid nodes (gamma probe: 500–1550 cps), whereas only three clear fluorescent hotspots within the nodes were visible. LiDAR-registered *fh*SPECT and NIR-fluorescence imaging was in concordance with conventional gamma probe and fluorescence imaging. **D**: Example of tracer-avid lymph node specimens in which overlying tissue caused fluorescent signal attenuation (nodes on the left), whereas LiDAR/*fh*SPECT clearly detected all tracer-avid hotspots

LiDAR-based surface scanning consistently provided high-resolution anatomical context, closely corresponding with white-light photography (Fig. 5).

Using intraoperative detection as applied reference standard, LiDAR/*fh*SPECT localised radioactive hotspots within all 38 tracer-avid LNs detected by gamma probe tracing. No radioactivity was localised in the 2 fluorescent-only nodes, which likewise lacked detectable radioactive signal on conventional ex vivo gamma probe assessment. No false-positive hotspots were observed in the 5 non-SNs. This resulted in an observed exploratory sensitivity of 95%, specificity of 100%, and accuracy of 96% with respect to the 40 tracer-avid LNs detected (Table 2).

LiDAR/NIR-fluorescence visualised all 40 fluorescent solitary LNs, in agreement with conventional ex vivo NIR-fluorescence imaging. Fluorescence signal could be spatially projected along 3D tissue contours derived from LiDAR-scanning (Fig. 2). Fluorescence signal intensity was influenced by tissue volume and depth. Signal attenuation was observed in lymph nodes covered by adipose tissue (Fig. 5D). Within the applied reference framework, LiDAR/NIR-fluorescence yielded exploratory sensitivity, specificity, and accuracy estimates of 100% in solitary lymph node specimens (Table 2).

#### ILND specimens (cN1 groins)

The 3 resected ILND specimens contained a total of 25 LNs, of which 6 tracer-avid hotspots had been identified on preoperative SPECT/CT (applied ILND reference framework). LiDAR/*fh*SPECT localised all 6 radioactive hotspots in concordance with preoperative SPECT/CT, whereas conventional ex vivo gamma probe assessment failed to detect 2 hotspots that demonstrated relatively low *fh*SPECT signal intensity and were located adjacent to more intense hotspots (case 10 & 14, ILND right; Table 1).

LiDAR/NIR-fluorescence localised 5/6 preoperatively identified hotspots. The addition of LiDAR did not improve hotspot detection by NIR-fluorescence compared with conventional ex vivo fluorescence imaging (see above). However, spatial co-registration of fluorescence images with LiDAR/*fh*SPECT revealed that the single missed fluorescent hotspot corresponded to a deeply located lymph node within the ILND specimen (case 14, ILND right; Table 1), suggesting fluorescence attenuation by overlying tissue.

### Histopathological correlation

Histopathological examination identified 6 tumour-positive LNs in 4 patients (mean diameter 12.3 mm; range 0.8–19 mm), including 2 metastatic LNs resected from cN0 groins (mean diameter 2.9 mm), and 4 metastatic LNs within the 3 resected ILND specimens from cN1 groins (mean diameter 17.0 mm).

**Fig. 6:**
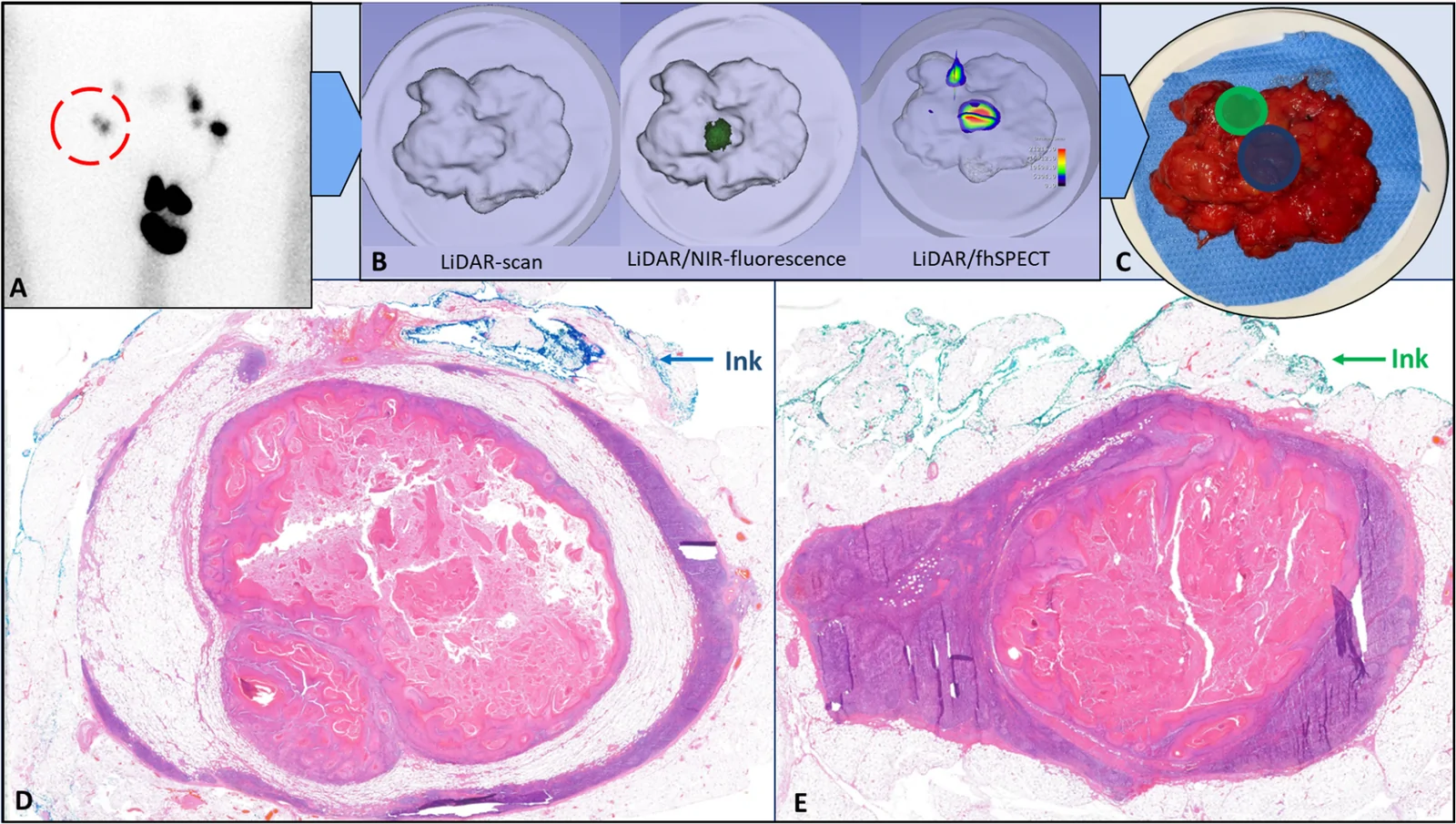
Ex vivo LiDAR-registered *fh*SPECT and NIR-fluorescence localisation of sentinel nodes within an inguinal lymph node dissection specimen, followed by pathological confirmation. Preoperative lymphoscintigraphy demonstrated two tracer-avid hotspots within the right (tumour-affected) groin (**A**). Following ILND, conventional gamma probe assessment and NIR-fluorescent imaging measurements identified one tracer avid hotspot within the specimen. Ex vivo hybrid specimen scanning (**B**), revealed only one corresponding fluorescent hotspot, whereas two distinct radioactive hotspots were seen using LiDAR/*fh*SPECT. Tracer-avid hotspots detected within the specimen were selectively inked blue and green (**C**). Subsequently, pathological processing (H&E)-stained whole-slide sections demonstrated penile squamous-cell cancer (SCC) involvement in both inked sentinel nodes, demonstrating spatial correspondence between hybrid ICG-^99m^Tc-Nanoscan tracer uptake identified by LiDAR/*fh*SPECT and histopathological findings (**D**, **E**). Overlaying tissue demonstrated fluorescent signal attenuation in the deeper located tumour-positive SN, and was therefore missed by NIR-fluorescence imaging. The remaining 8 non-tracer-avid lymph nodes found within the ILND specimen were tumour negative

All tumour-positive LNs corresponded to tracer-avid hotspots identified preoperatively and were visualised using LiDAR/*fh*SPECT. LiDAR/NIR-fluorescence imaging did not visualise the deeply located tumour-positive LN within the third ILND specimen (case 14; Fig. 6). All tumour-positive nodes within the ILND specimens had also been identified preoperatively using [¹⁸F]FDG-PET/CT, consistent with previous reports describing concordance between lymphatic mapping and metabolic imaging findings [28].

Ex vivo gamma probe count rates were numerically lower in metastatic compared with non-metastatic lymph nodes (mean: 1205 vs. 4596 cps; Mann–Whitney U test, *p* = 0.76). No clear association was observed between ex vivo gamma probe count rates and metastatic diameter. Although the limited number of involved nodes precludes firm interpretation of these findings, they are consistent with previous hybrid tracer studies [29].

## Discussion

While intraoperative molecular imaging techniques are designed to sensitively detect tracer uptake, they generally lack the spatial context required to localise signals within 3D anatomy or complex tissue specimens. In the present study, LiDAR provided spatially augmented 3D localisation of hybrid-tracer uptake for *fh*SPECT and NIR-fluorescence imaging of nodal specimens. The high degree of agreement between LiDAR-registered and conventional detection techniques in solitary tracer-avid LNs likely reflects their spatial accessibility and the limited amount of surrounding tissue capable of attenuating NIR-fluorescent signals [30, 31]. The added value of the 3D surface registration became most apparent in ILND specimens, where LiDAR/*fh*SPECT enabled localisation of tracer-avid nodes that were incompletely resolved using conventional ex vivo assessment. These findings support the feasibility of integrating handheld LiDAR scanning into specimen-oriented molecular imaging workflows, particularly in more anatomically complex specimens.

A key challenge during surgical nodal staging is identifying the “nodes that matter” while preserving uninvolved lymphatic anatomy. Although SN procedures are guideline-recommended for clinically node-negative groins, ILND remains standard treatment in node-positive groins, or in institutions where SN procedures are not available [2]. Harvesting larger numbers of LNs may increase the likelihood of tumour detection but is also associated with substantial morbidity and may result in overtreatment [32]. Imaging technologies that improve spatial interpretation of tracer uptake within complex nodal specimens may therefore be of future interest, potentially helping to balance oncological completeness with preservation of healthy tissue. However, while preoperative SPECT/CT represented the most appropriate available anatomical reference for the tracer-avid hotspots within the ILND specimens, exact one-to-one spatial correspondence with ex vivo hotspot localisation cannot be assumed because of tissue deformation, specimen manipulation, and changes in specimen orientation following resection. The present ex vivo workflow helps to address some of these challenges. Although the current data are insufficient to support changes in surgical decision-making or nodal management strategies, they suggest that more detailed assessment of ILND specimens in routine care may improve documentation of nodal tumour spread.

Interpretation of hotspot localisation requires consideration of signal scaling and modality–specific detection characteristics inherent to molecular imaging. Threshold adjustment required optimisation on a per-patient basis. Across all nuclear imaging settings (preoperative, intraoperative, and ex vivo), hotspot identification relied on thresholding; consequently, variations in tracer uptake influenced the visibility of low-intensity hotspots, particularly in patients with marked heterogeneity in nodal tracer uptake (see Fig. 5A). A notable example requiring substantial threshold adjustment is the well-known “overshining” phenomenon, whereby high-intensity radioactive hotspots (e.g. the injection site) may mask adjacent low-activity hotspots, thereby reducing their visibility during hotspot localisation [15]. In contrast, NIR-fluorescence imaging is not affected by this “overshining” phenomenon due to its superficial detection characteristics. However, its limited tissue penetration may hamper hotspot detection owing to signal attenuation in deeper or adipose-covered tissue, as illustrated by the missed deeply located metastatic LN in the ILND cohort (Fig. 6). These discordant findings reflect complementary modality-specific strengths and limitations rather than superiority of one modality over the other. Parallel research efforts are focusing on standardising quantitative signal-to-background interpretation and threshold selection, which may improve reproducibility and reduce operator dependence in future implementations [33].

Freehand SPECT has previously demonstrated value in intraoperative radioguidance by enabling volumetric localisation of radiotracer uptake beyond the point-based count-rate measurements provided by conventional gamma probes [10–15, 34, 35]. However, most currently available intraoperative molecular imaging approaches still provide limited anatomical context, relying on point-based signal detection or 2D anatomical overlays [34, 36]. Anatomical co-registration approaches, including fusion of *fh*SPECT with ultrasound, have further illustrated the value of integrating radioactive signal information with anatomical context [37]. LiDAR-based 3D surface scanning offers a non-ionising alternative in the form of anatomical surface reconstruction and provides a practical extension of this concept for specimen-oriented molecular imaging workflows. Prior studies have demonstrated the feasibility of combining LiDAR with *fh*SPECT for spatial localisation of PSMA-avid prostate cancer specimens, focussing on radioactive signal co-registration alone [21, 22]. The present work extends this concept to hybrid radio- and fluorescence-guided nodal specimen assessment. This hybrid imaging concept introduces new challenges related to modality-specific signal attenuation, with these effects becoming increasingly relevant in larger and more complex specimen anatomy. A recent minimally invasive use case further illustrates the broader potential of such spatially corrected augmented reality visualisation concepts [38].

From a workflow perspective, the presented ex vivo imaging strategy requires additional infrastructure and operator expertise. LiDAR acquisition took approximately 1–2 min per specimen tray, *fh*SPECT acquisition required 2–3 min, and fluorescence imaging took less than 1 min, resulting in a total acquisition and post-processing time of approximately 10 min per specimen tray. While these steps were manageable in an ex vivo research setting, integration into routine intraoperative nuclear medicine workflows would require further refinement in terms of hardware and software. In the near term, this approach may support specimen orientation during pathological evaluation in centres where radioguided or hybrid tracer-guided surgery is routinely performed. More broadly, this work establishes a framework for exploring how spatially augmented molecular imaging may be integrated into future surgical workflows.

This study was not designed or powered to formally evaluate diagnostic performance. The cohort size was limited (28 groins; 48 specimens), including only 3 ILND specimens and 6 tumour-positive LNs, resulting in exploratory performance estimates that cannot be generalised. Furthermore, imaging was performed in a controlled ex vivo environment, allowing stable specimen positioning and precise spatial co-registration, but limiting direct extrapolation to intraoperative in vivo settings. Consequently, these findings should be considered hypothesis-generating rather than supporting immediate clinical implementation or changes in surgical management. Future prospective studies are needed to validate these findings in larger cohorts, establish standardised imaging workflows, and quantitative thresholds, and determine whether spatially augmented imaging improves intraoperative interpretation, workflow efficiency, and clinical decision-making.

## Conclusions

Handheld LiDAR-registered 3D surface reconstruction provides anatomical context for *fh*SPECT and NIR-fluorescence imaging, thereby facilitating spatial localisation of hybrid tracer uptake within nodal specimens resected from penile cancer patients. These findings provide a framework for future studies exploring this form of spatially augmented intraoperative molecular imaging in vivo.

## Data Availability

The datasets generated and/or analyzed during the current study are available from the corresponding author on reasonable request.

## Abbreviations

BMI: body mass index
Cps: counts per second
CT: computed tomography
FDG: fluorodeoxyglucose
fhSPECT: freehand single–photon emission computed tomography
FNAC: fine–needle aspiration cytology
H&E: haematoxylin and eosin
ICG: indocyanine green
ILND: inguinal lymph node dissection
IQR: interquartile range
LiDAR: light detection and ranging
LN: lymph node
MBq: megabecquerel
NIR: near–infrared
PET/CT: positron emission tomography/computed tomography
SD: standard deviation
SN: sentinel node
SPECT/CT: single–photon emission computed tomography/computed tomography
